# Urine can speed up the re-epithelialization process of prostatic urethra wounds by promoting the proliferation and migration of prostate epithelial cells

**DOI:** 10.1007/s11255-018-2019-2

**Published:** 2018-11-07

**Authors:** Lixin Wang, Ying Cao, Ye Tian, Guangheng Luo, Xiushu Yang, Zhaolin Sun

**Affiliations:** 1 Guizhou Provincial People’s Hospital, Guiyang, Guizhou China; 2 Guizhou Medical University, Guiyang, China

**Keywords:** Urine, Benign prostatic hyperplasia, Re-epithelialization

## Abstract

**Objectives:**

The present study aimed to investigate the influence of urine on re-epithelialization in canine prostatic urethra after prostatectomy and explore possible causes.

**Method:**

We established two groups of prostatic canine models. The first group contained urine that canines underwent the surgery by two-micron laser resection of the prostate-tangerine technique (TmLRP-TT), and no transurethral catheter was required. The second group was without urine that canines accepted the surgery by TmLRP-TT add ureter skin ostomy urine bypass. Histopathology of re-epithelialization of repair in trauma in canine prostatic urethra was observed by hematoxylin and eosin (HE) staining, and immunochemistry was used to determine the expression of transforming growth factor-β_1_ (TGF-β_1_). Human prostate epithelial line (BPH-1) cells were cultured with or without urine and the abilities of proliferation and migration were tested by CCK-8 and transwell assays, respectively.

**Results:**

The histology displayed that there was distinct proliferation of prostatic cell under the wound after 3 days, re-epithelialization began after 9 days, and finished after 28 days at urine group. The TGF-β1 like-IR in prostatic epithelium cells and fibroblast cells under the wound at urine group were strikingly increased as compared with the cells at no urine group after 3, 9, and 11 days, respectively (*p* < 0.05). In CCK-8 and Transwell assays, an increase of cells’ proliferation and migration was detected in urine culture group compared with no urine culture group (*p* < 0.05).

**Conclusion:**

Urine may speed up the re-epithelialization process for prostatic urethra wounds by promoting proliferation and migration of prostate epithelial cells.

## Introduction

Benign prostatic hyperplasia (BPH) is a common disease affecting the quality of life of senile male [1]. Approximately 20% of all BPH patients with symptomatic disease eventually undergo surgery [2]. With promotion and application of laser technology in urology in recent years, two-micron laser resection of the prostate-tangerine technique (TmLRP-TT) is becoming a new minimally invasive procedure for treatment of BPH [3, 4]. Urothelial prostatic urethra self‑healing, also called re‑epithelialization, is the fundamental process of wound healing following injury and facilitates the surgical wound closure to reduce complications, such as postoperative hemorrhage, urinary tract infection, and uncomfortable postoperative symptoms, including urinary frequency, urgency, and urodynia. The traditional concept that prevails among urologists is that re-epithelialization of the prostatic urethra results from migration and differentiation of proliferating epithelial cells from the edges of the wound at the bladder neck after injury analogous to skin wound repair. However, Pow-Sang et al. [5], Orihuela et al. [6] and our previous studies [7, 8] have confirmed that the re‑epithelialization of the prostatic urethra after utilizing TmLRP-TT in canine prostate models may result from proliferation, migration, and differentiation of prostatic basal cells from residual prostate tissue under the wound.

Patients with BPH were routinely left with a three-chamber airbag urethral catheter for bladder rinsing after surgery, and the prostatic fossa also could be compressed to achieve hemostasis. However, a three-chamber balloon urethral catheter not only restricts the patient’s activity and causes the patient to be unwell, but also increases the opportunity of urinary tract infection. The time for the removal of the urethral tube after surgery has not been standardized. Urethra is the output channel of urine. After prostatectomy, urine inevitably reaches prostatic urethral wound in the prostate. Whether urine will affect the process of re‑epithelialization of prostatic urethra after BPH surgery has not been yet reported. In this study, we established two types of canine models (urine group and no urine group) to investigate the influence of urine on re-epithelialization in canine prostatic urethra after prostatectomy. In addition, we observed proliferation and migration of BPH-1 cells with or without urine cultured in vitro by CCK-8 and transwell migration assays, in order to provide useful experimental basis for early removal of urethral catheter in clinic.

## Materials and methods

### Canines

Twenty-four healthy adult male crossbred canines were obtained from Zunyi Medical College (Zunyi, Guizhou province, China). The animal models were approved by Medical Ethics Committee of Guizhou Provincial People’s Hospital. The animals were 5–7 years old and weighed 18–22 kg.

### Modeling of two-micron laser resection of the prostate

All operations were performed using the same two-micron continuous wave laser Tm: YAG laser system (RevoLix; Lisa Laser Products, Katlenburg, Germany). The wavelength of laser was 2.013 µm and the energy was transmitted at 70 W of power output through a flexible 550 µm diameter fiber. After general anesthesia was achieved with 10% chloral hydrate (0.003 ml/g), the canine was placed in the supine position on an operating table. The lower abdomen was entered through a medial and longitudinal incision and the anterior wall of the bladder was freed. A purse suture was performed in the anterior wall of the bladder, an incision was made within the purse to allow the placement of a 26F continuous-flow resectoscope, and then the suture was fastened. Under saline irrigation, a resectoscope was placed into the prostatic urethra through the internal urethral orifice. The laser vaporization of the prostate was applied in patients as the same, as previously described [9, 10]. During the vaporization, the fiber was continuously swept in half-moon mode to resect all prostatic urethra and the majority of the prostatic tissues, while avoided injury to the prostatic capsule. No transurethral catheter was required as well.

It was attempted to clarify whether urine can affect the process of re-epithelium after operation of BPH. Twelve canines underwent the surgery of ureter skin ostomy urine bypass. The specific actions were as follows: the lower abdomen longitudinal incision was about 2.5 cm, which found in the bladder in both sides of the ureter, the distal end of ureter cut ureter, was free about 3 cm in the bilateral lower abdomen incision which was about 0.5 cm, the bilateral ureter from the incision, and skin incision anastomosis, complete ureter skin mouth, close abdominal incision.

### Histopathologic examination

Twenty-four canines were randomly divided into urine group (12 canines) and no urine group (12 canines). In urine group, the canines underwent the surgery by TmLRP-TT, and no transurethral catheter was required. In no urine group, the canines underwent the surgery of ureter skin ostomy urine bypass. Canines in urine group and no urine group were randomly divided into four subgroups (3, 9, 11, and 28 days after surgery) and each subgroup contains three canines. The canines of each subgroup were sacrificed, and wound specimens from the prostatic urethra were harvested and fixed in 4% formalin at 3, 9, 11, and 28 days after laser treatment. Prostate tissue samples were cut in the transverse plane at the level of the mid prostatic urethra to permit inspection of the lesion. After embedding in paraffin, slides (4 µm) were histologically examined by hematoxylin and eosin (HE) staining.

### Immunohistochemistry staining

Immunohistochemical staining was performed as described earlier [11]. Briefly, the sections were treated with blocking buffer (Dako Denmark A/S, Glostrup, Denmark) for 30 min at room temperature (RT) and thereafter incubated with transforming growth factor-β1 (TGF-β_1_) antibody (1:200 dilution in Tris–NaCl buffer; Abcam, Cambridge, UK) overnight at 4 °C. Following a thorough rinse in Tris–NaCl buffer, the sections were incubated with biotinylated goat anti-rabbit IgG (1:200 dilution in Tris–NaCl buffer; Abcam, Cambridge, UK) for 60 min at RT. Sections were subsequently incubated with avidin-biotinylated enzyme complex (ABC) and DAB, and then dehydrated with increasing the concentrations of ethanol, cleared with xylene, and mounted in permount. Negative controls for these immunohistochemically procedures were incubated with non-immune serum instead of the primary antibodies, which resulted in no detectable staining. The optical densities (OD) of TGF-β_1_ like immunoreactivity (IR) in prostatic urethra wound from 3 to 11 days after utilizing TmLRP-TT were measured by using a CM2000B Biomedical Image Analysis System (Beihang University, Beijing, China). The OD of TGF-β_1_ like-IR was analyzed by micro densitometry in prostatic epithelium cells and fibroblast cells under the wound. Five random fields of interest were measured and the OD measurements were averaged as well.

### Cell line, collection, and treatment of urine specimen

Human prostate epithelial line (BPH-1) cells were obtained from ATCC (USA). Cells were cultured in Dulbecco’s modified Eagle’s medium (DMEM; Gibco, Grand Island, NY, USA) with 10% fetal bovine serum (FBS; Gibco, Grand Island, NY, USA) and incubated at 37 °C in an incubator with 5% CO_2_. After obtaining the consent of the patient and his family, it was attempted to collect the urine of the first day after the operation for BPH patients who underwent prostatectomy. The urine specimens were collected in the biological super Net Taichung using sterile filter, and the filtered specimens were gathered by sterile centrifuge tubes and marked number. The cell group was divided into no urine culture group and urine culture group; no urine culture group used DMEM medium, and incubated at 37 °C in CO_2_ culture box culture cell; urine culture group in DMEM medium added aseptic urine and incubated at 37 °C in CO_2_ culture box culture cell.

### CCK-8 experiment

The urine sample, which was treated well, was diluted with medium and urine, and the urine cell culture group with dilution of multiples of 2, 4, 8, 16, 32, 64 times was prepared. 12 h later, the 96-well cell plate was removed from the incubator. The old medium in the hole was discarded. Each urine specimen in the experimental group and the control group was provided with 3 complex holes in each group. In the experimental group, the whole of medium of mixed urine was replaced, and the control group was replaced by the complete medium of equal volume. It was attempted to put it back in the incubator and continued to incubate for 24 h. After the culture was finished, the 10:1 proportion mixed medium Cell Counting Kit-8 (Dojindo Biochem, Shanghai, China) was used, each hole joins the 110 µl, and the attention adds the sample process avoiding the bubble to produce. It was then put back in the incubator at constant temperature to be incubated for 2 h. The cell viability was then detected by using a microplate reader at wavelength of 450 nm.

### Transwell experiment

Cell migration assay was carried out using Transwell Permeable Support (Corning Incorporated, Corning, NY, USA). Control cells, urine culture group BPH-1 cells, and no urine culture group CBPH-1 cells were carefully transferred on the top chamber of each transwell apparatus at a density of 1 × 10^6^/ml. Cells were allowed to migrate at 37 °C for 24 h. Cells that had penetrated to the bottom side of the membrane were then fixed in formalin, stained using 0.1% crystal violet solution (Beijing Solarbio Technology Co. Ltd, Beijing, China) and observed under a microscope. The cells were seeded in 200 µl serum-free media into the upper wells, which were previously coated with Basement Membrane Extract (BME) and 500 µl of media into the bottom wells. After 24 h of CO_2_ incubation at 37 °C, the invasive cells on the bottom surface of the membrane were fixed and stained using 0.1% crystal violet solution. Cells were counted in five randomly selected fields.

### Statistical analysis

In the present study, SPSS 22.0 software (SPSS Inc., Chicago, IL, USA) was used for performing all statistical analyses, and statistical data are presented as mean ± standard deviation (SD). The statistical differences were evaluated using the independent-samples t-test statistical analysis, and *p* < 0.05 was statistically considered significant.

## Results

### Histopathological changes of re-epithelialization in canine prostatic urethra

**Fig. 1:**
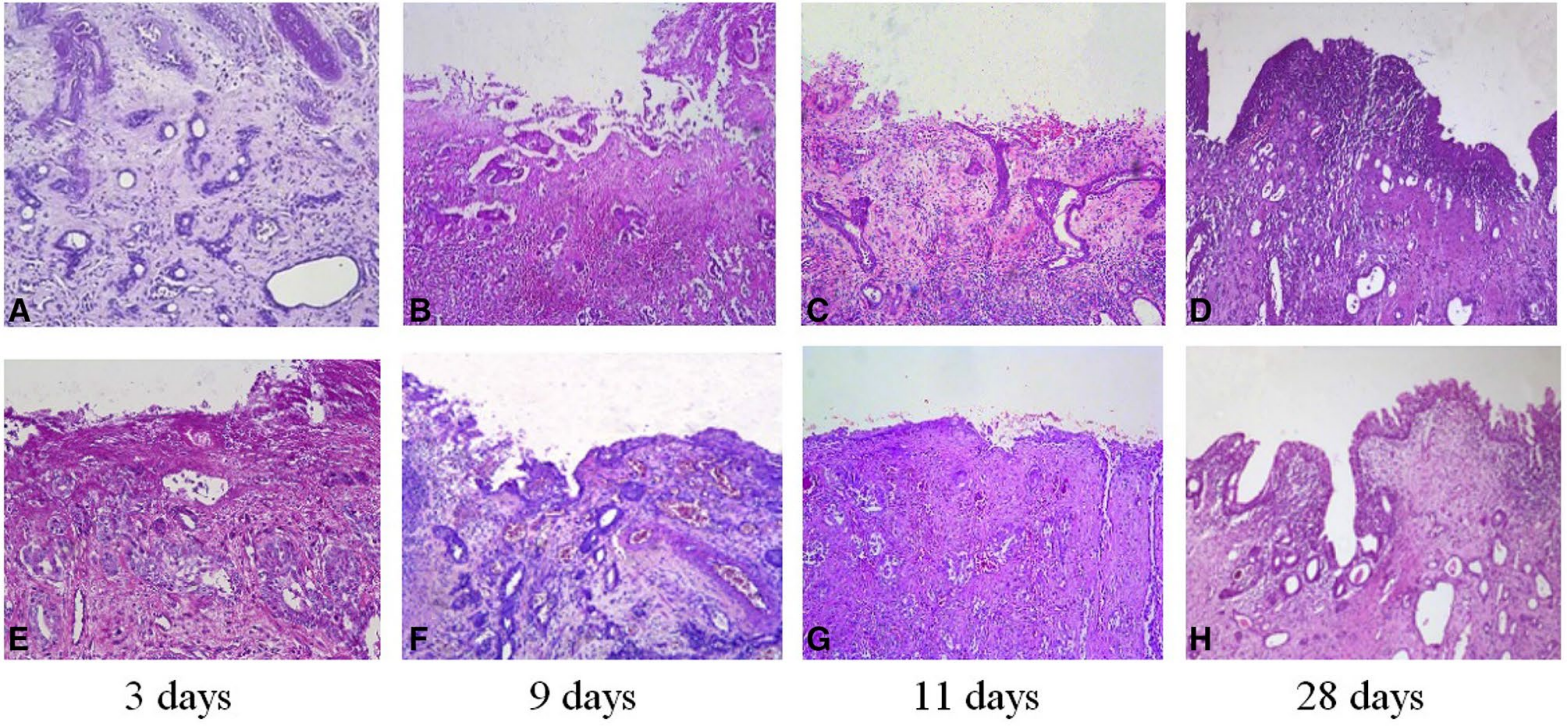
Histopathological changes of re-epithelialization in canine prostatic urethra in no urine group and urine group (HE × 200). **a**–**d** no urine group; **e**–**h** urine group

In no urine group, 3 days after laser irradiation, a zone of coagulation necrosis and acute inflammatory exudate on the wound surface was evident, and we could not observe proliferating epithelial cells from residual prostatic epithelium **(**Fig. 1a**)**. At 9th day, there was a prominent proliferation of the epithelium originating from the underlying prostatic epithelium. This proliferation epithelium had a tendency to migrate to the wound surface, but no regenerated epitheliums were observed on the wound surface **(**Fig. 1b**)**. Then 11 days after surgery, this germinating epithelium extended in some areas to the lumen of the cavity with focal re-epithelialization **(**Fig. 1c**)**. Next, 28 days after operation, the majority of the wounds were covered with regenerated epithelium. The regenerated epithelium was thick 6–7 layer, and replaced by transitional epithelium with a distinct layer of umbrella cells, suggesting the urine differentiation. However, there are still several special treatment wounds which haven’t observed the regeneration epithelium cover **(**Fig. 1d**)**.

In urine group, there was a distinct proliferation of prostatic cell under coagulation necrosis at 3 days after surgery **(**Fig. 1e**)**. Then, 9 days after laser radiation, histological findings showed distinct prostatic epithelial proliferation under coagulation necrosis. In some areas the proliferation of epithelium was extended to the lumen of the cavity with a focal re-epithelization of the surface which demonstrated that the re-epithelialization was begun **(**Fig. 1f**)**. Next, 11 days after laser radiation, regenerative epitheliums of the wounds surface were increased **(**Fig. 1g**)**. After 28 days, all wounds were covered with regenerated epithelium, and the regeneration epithelium was polar, and the surface of the umbrella cells was observed as well **(**Fig. 1h**)**.

Histopathological changes of re-epithelialization in canine prostatic urethra in no urine group and urine group are shown in Fig. 1.

### Expression of TGF-β_1_ at prostatic urethra wound

The positive expression of TGF-β_1_ was observed in prostatic epithelial cells and fibroblast cells. The TGF-β_1_ like-IR in prostatic epithelial cells and fibroblast cells under the wound in urine group were strikingly increased compared with these cells in no urine group at 3, 9, and 11 days. Levels (OD) of TGF‑β_1_ like-IR in prostatic epithelial cells and fibroblast cells at the prostatic urethra wound during 3 to 11 days after surgery are shown in Table 1.

**Table 1:** Levels (OD) of TGF‑β_1_ like-IR in prostate epithelial cells and fibroblast cells at the prostatic urethra wound during 3–11 days after surgery

	Prostate epithelial cells		Fibroblast cells	
	No urine group	Urine group	No urine group	Urine group
At 3 days	0.15 ± 0.03	0.24 ± 0.05*	0.14 ± 0.05	0.23 ± 0.03*
At 9 days	0.14 ± 0.04	0.26 ± 0.03*	0.13 ± 0.04	0.25 ± 0.04*
At 11 days	0.17 ± 0.05	0.26 ± 0.02*	0.15 ± 0.03	0.26 ± 0.04*

Data are presented as mean ± SD

*p < 0.05 as compared to no urine group

### CCK-8 experiment to detect the proliferative ability of BPH-1 cells

**Fig. 2:**
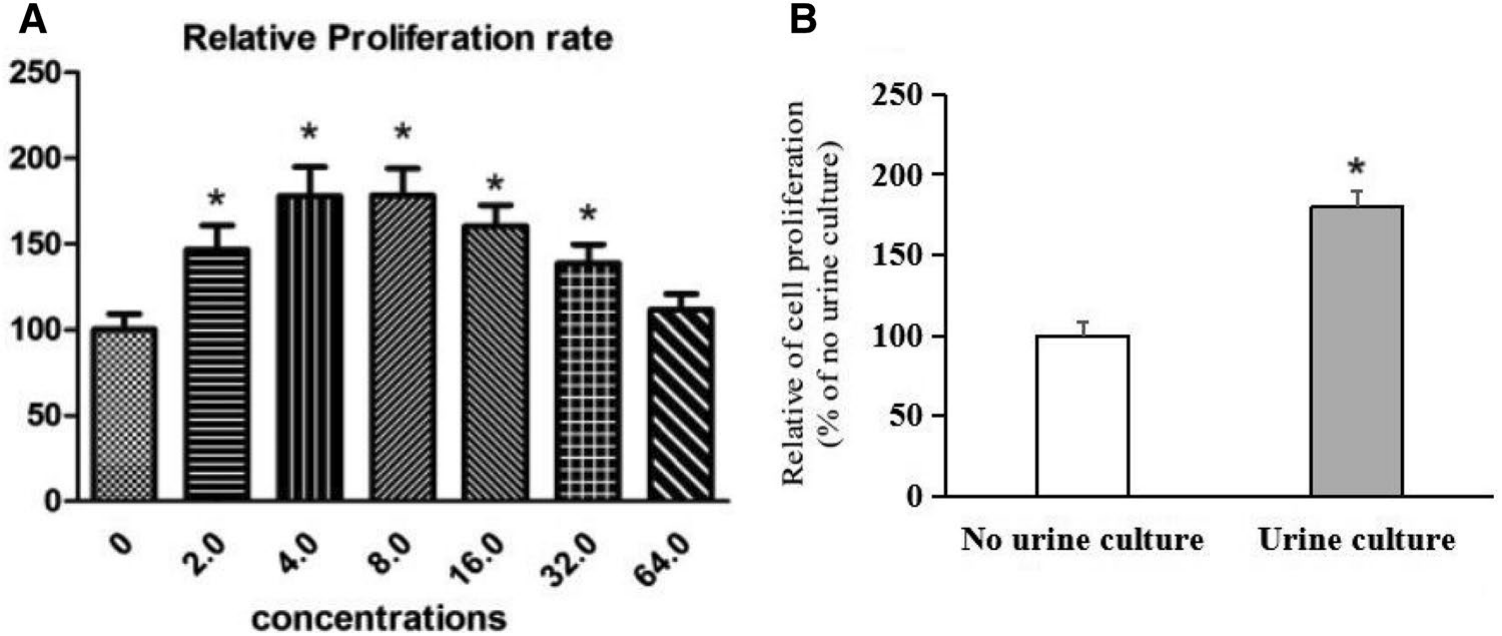
The effect of urine on proliferative capacity of BPH-1 cells. **a** Effects of different concentrations of urine in medium on proliferation of BPH-1 cells; **b** comparing the effects of the optimal concentration of urine culture on the proliferative capacity of BPH-1 cells. Data are presented as mean ± SD. *p < 0.05 as compared to no urine group (the concentration of urine is 0)

Here, CCK-8 experimental results showed that with decrease of the dilution of urine concentration, proliferative ability of BPH-1 cells in a certain low concentration range (2.0 ≤ *c* ≤ 32.0) gradually increased. When the dilution of urine is 64 times, its proliferative capacity had no statistical difference as compared with no urine culture group (*p* > 0.05). Compared with no urine culture group, when dilution is carried out 4 times, proliferative activity of BPH-1 cells is higher than no urine group cell activity (*p* < 0.05), in this concentration, promotion of proliferation of BPH-1 cells is more obvious **(**Fig. 2a**)**. Therefore, we selected 4 times dilution as the next experimental group with the optimal concentration of urine medium and transwell migration assays. Compared with no urine culture group, the cell proliferation activity of the urine culture group was significantly enhanced with 4 times dilution, and the difference between the two groups was statistically significant (*p* < 0.05). The effect of urine on proliferative capacity of BPH-1 cells is shown in Fig. 2b.

### Transwell experiment to detect the migration ability of BPH-1 cells

**Fig. 3:**
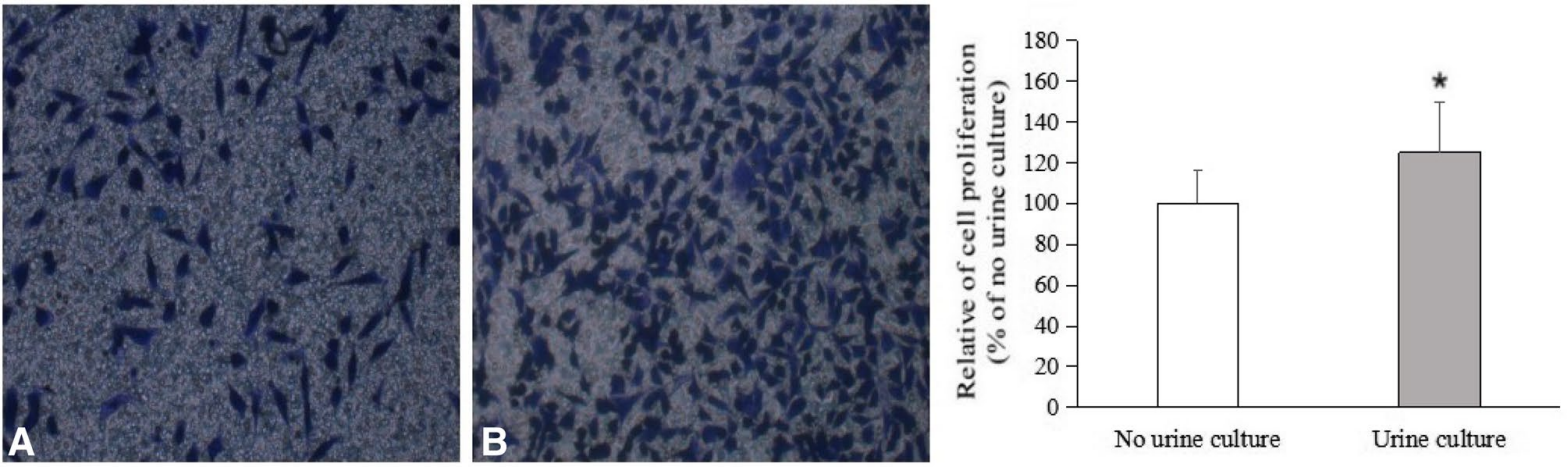
The effect of urine on migration capacity of BPH-1 cells. **a** No urine group; **b** urine group. Data are presented as mean ± SD. *p < 0.05 as compared to no urine group (the urine concentration is 0)

The achieved results showed that BPH-1 cells in both no urine culture group and urine culture group could be filtered through the Transwell compartment, and the number of cells in urine culture group was significantly increased compared with no urine culture group (*p* < 0.05). The above-mentioned results suggested that urine increased the migration capacity of BPH-1 cells. The effect of urine on migration capacity of BPH-1 cells is shown in Fig. 3.

## Discussion

As the output of urine, urine will inevitably reach the prostatic urethra wound after prostatectomy. Urine is a metabolic end product of the body, and it is used for the diagnosis and prognostic judgment of urogenital diseases in clinical trials. However, the possible role of urine in tissue repair has been rarely reported. It has been proved that some metabolites in urine can affect the survival and proliferation of cells. For instance, uric acid has a significant effect on the proliferation of fibroblast cells [12].

Our previous studies have confirmed that the re-epithelialization of the prostatic urethra resulted from the migration and differentiation of proliferated prostatic basal cells from residual prostatic epithelium under the wound after utilizing TmLRP-TT in a canine prostate model [8]. In the current study, TmLRP-TT (as urine group) in 12 canines and TmLRP add ureter skin ostomy urine bypass (as no urine group) in 12 canines were accordingly conducted. We harvested prostatic urethra wound specimens for HE staining at 3, 9, 11, and 28 days after operation to observe the histopathological changes of re-epithelialization under light microscope. The results showed that re‑epithelialization of prostatic urethra wound could be completed through both migration and differentiation of proliferated prostate basal cells from residual prostatic epithelium under the wound in urine group and no urine group. That is to say, in presence or absence of urine, the wound surfaces all can complete the re-epithelialization of the wound. However, compared with no urine group, the proliferation and migration of prostatic basal cell under the wound were observed earlier and the re-epithelialization was faster in urine group, suggesting that urine could accelerate the re-epithelialization process.

Numerous researches confirmed that cytokine plays an important role in the regulation of wound healing events. TGF-β_1_ is an important cell growth factor playing a significant role in wound healing process [13]. A previous study has shown that inflammatory cells, keratinocytes, and fibroblasts can secrete TGF-β_1_ to maintain the high TGF-β_1_ concentration and promote local inflammatory reaction, accelerate the proliferation of wound granulation tissue and the process of re-epithelialization in the early and middle stages of skin wound repair [14]. The ability of TGF-β_1_ to directly accelerate wound healing has been shown in rat incisional wounds [15, 16]. Exogenous application of TGF-β_1_ to skin wounds also enhanced epithelial regeneration in vivo [17, 18]. In order to observe whether the presence or absence of urine affects the expression level of TGF-β_1_ in prostatic urethral wound, we investigated the expression level of TGF-β_1_ by immunohistochemical staining method. We found that there were enhanced TGF-β_1_ expressions in prostatic epithelium cells and fibroblast cells under the wound in urine group compared with the cells at no urine group at 3, 9, and 11 day after surgery. These results demonstrated that in the presence of urine, wound was at a higher level of TGF-β_1_ expression, by autocrine and paracrine, direct or indirect, individual or collaborative trials to speed up the proliferation and migration of prostatic basal cell, as well as rapidly initiating the process of re-epithelialization of wound healing.

Based on in vivo experiments, we found that with or without urine, the wounds were covered with regenerated epithelium which was replaced by transitional epithelium with a distinct layer of umbrella cells at 28 days after surgery. This phenomenon revealed that the presence or absence of urine in the wound does not affect the differentiation of regenerated epithelium into transitional epithelium. Thus, we speculated that urine may speed up the re-epithelialization process by promoting the proliferation and migration of. Therefore, we observed the effect of urine on proliferation and migration of BPH-1 cells in vitro by CCK-8 and transwell assays, respectively. The results of CCK-8 and transwell experiments showed that the proliferation and migration of BPH-1 cells in urine culture group was significantly increased compared with no urine culture group. According to in vivo research’s results, we found that urine can accelerate the process of re-epithelialization of prostatic urethra wound surface by promoting the rapid proliferation and migration of prostatic basal cells under the wound.

Urine is normally germ-free fluid consisting of 91–96% water and an assortment of dissolved substances [19], including inorganic salts (Na^+^, K^+^, Cl^−^, Ca^2+^, sulfate, phosphate, ammonium, etc.) and organic compounds (urea, uric acid, urokinase, growth hormone, erythropoietin, gonadotropin, callicrein, antitumor peptides, growth factors, etc.). It is not clear which components of urine could accelerate the process of wound re-epithelialization; thereby further studies are required in vitro. According to the achieved results, we expressed that in terms of treatment effects, the urinary catheter should be removed as early as possible in BPH patients after operation in order to shorten the time of urine tube indwelling, as well as alleviating patients’ discomfort, reducing the postoperative complications (e.g., infection), and also can accelerate the re-epithelialization process.
